# Treatment of menstrual migraine; multidisciplinary or mono-disciplinary approach

**DOI:** 10.1186/s10194-017-0752-z

**Published:** 2017-04-17

**Authors:** Hester Witteveen, Peter van den Berg, Guus Vermeulen

**Affiliations:** 1 Isala, Department of Neurology, Dr van Heesweg 2, 8025 AB Zwolle, The Netherlands; 2 Isala, Department of Gynaecology, Dr van Heesweg 2, 8025 AB Zwolle, The Netherlands

**Keywords:** Menstrual migraine, Menstrual related migraine, Hormonal treatment, Multidisciplinary approach

## Abstract

**Background:**

The aim of this study was to compare a multidisciplinary approach of menstrual (related) migraine, combining the neurological and gynaecological consultation, to a mono-disciplinary approach involving neurological treatment.

There is a clear relationship between the menstruation cycle and the occurrence of migraine (menstrual migraine). Nowadays the treatment of menstrual (related) migraine is performed by a neurologist. A treatment with attention to hormonal treatment seems more convenient.

**Methods:**

This retrospective study was performed in a cohort using data of 88 women with menstrual (related) migraine who visited the menstrual migraine clinic between 2012 and 2014 (intervention group). The results were compared to a historical control group, which consisted of women with menstrual (related) migraine who were treated before 2012 and received a mono-disciplinary approach.

**Results:**

In the intervention group the Headache Impact (HIT) score significantly improved (65 to 59 points). The mean headache days per month declined significantly (from 6 to 3.83 days) and these women needed less use of pain medication. In the control group the decline in HIT score was less striking (65 to 63.5 points) and the mean headache days per month increased (6 to 6,5 days). It appeared that 20 out of 27 patients in the control group required a gynaecological consultation in course of time.

**Conclusion:**

A multidisicplinary treatment of women with menstrual (related) migraine gives better results compared to a mono-disciplinary approach. These results should be interpreted with caution as we performed a retrospective study with a relative small control group.

## Background

Migraine is a common headache disorder. There is a clear relationship between the menstruation cycle and the occurrence of migraine (menstrual migraine). Studies show that about 50% of the women with migraine experience a relationship between menstruation and the occurrence of migraine attacks [1–3]. The menstrual migraine attacks are usually without aura [4, 5], last much longer, are more often associated with severe nausea and are more painful than non-menstrual attacks [6].

Menstrual migraine is usually treated as regular migraine. Clinical studies also show the possibility of a short-term prevention of perimenstrual migraine with triptans or hormonal treatment [7, 8]. Recently, non-invasive vagus nerve stimulation has been suggested as prophylactic treatment for menstrual (related) migraine [9]. The majority of migraine patients is treated in primary care. If needed, patients are referred to the neurologist for further treatment. Patients with menstrual migraine also can be referred for hormonal treatment to a gynaecologist.

In this study we investigated if a multidisciplinary approach, combining the neurological and gynaecological consultation, contributed to an improved treatment of menstrual (related) migraine, compared to treatment only by a neurologist.

## Methods

### Study setting

Patients with migraine in primary care, were referred by their general practitioner to the headache clinic of the neurology department, Isala hospital Zwolle, for further treatment. Before 2012, women with menstrual (related) migraine were treated only by the neurologist at the headache clinic. Starting in 2012, women who were suspected of suffering from menstrual (related) migraine at the headache clinic were referred to the menstrual migraine clinic (MMC). All women who visited the MMC between March 2012 and December 2014 were included in this study. During the first appointment at the MMC, headache diaries were reviewed to determine if the migraine attacks were related to menstruation. Inclusion criteria were women with (probable) menstrual or menstrual related migraine according to the ICHD-III criteria. Exclusion criteria were depression, chronic migraine or medication overuse headache according to the ICHD-III criteria [4].

### Menstrual migraine clinic

The difference between the headache clinic and the menstrual migraine clinic is a multidisciplinary approach. At the MMC the patient is seen at the same time by a nurse practitioner specialised in migraine who initiates migraine treatment such as attack and prophylactic medication, and a gynaecology nurse practitioner who focuses on prevention of the hormonal trigger of menstrual migraine through hormonal treatment. Drug therapy was individualised to the patient’s needs according to the guideline for headache of the Dutch Association of Neurology [10]. Preventing a decline in estrogen level was mostly done by treatment with Cerazette (contraception pill with progestogens only) to avoid hormonal fluctuations and to achieve an amenorrhoic situation. In contrast to a combined oral contraceptive pill, Cerazette (containing only progesterone) is not associated with an increased risk of venous thromboembolism and ischemic stroke [11]. Other hormonal interventions were continuous usage of an oral contraception pill during several months, placing of an intra-uterine device (IUD), or using estradiol patches around menstruation. Evaluation took place after 3, 6 and 9 months (3 follow-up moments). During these follow-up moments treatment was evaluated, and adjusted if necessary.

### Intervention group versus control group

The intervention group consisted of women with menstrual (related) migraine who were treated at the MMC between 2012 and 2014 and received multidisciplinary care (*N* = 88).

The control group consisted of women with menstrual (related) migraine who were treated at the headache clinic before 2012. They were treated by a neurologist and a migraine nurse practitioner (*N* = 27). These patients received questionnaires to evaluate the mono-disciplinary treatment (number of responders = 22). The number of visits at the outpatient clinic was similar for both groups.

### Data collection

The database contained per patient five contact moments: Baseline (visit at the headache clinic at the department of Neurology), the first visit at the MMC and the follow-up moments after 3, 6 and 9 months at the MMC. During these visits the patients filled in questionnaires. One of these questionnaires was about lifestyle, headache and demographic characteristics. The patient also filled in a Headache Impact Test-6 and a SF12 Health Survey.

### Statistical analysis

The statistical analysis of the data was done using the program SPSS for Windows, Version 22.0 (IBM SPSS Statistics, IBM Corporation, Armonk, NY). At first the descriptive statistics were performed. Comparison of nominal variables between both groups was done with a 2x2 cross table and the Fisher’s exact test. Before analysing the inferential statistics the variables were checked for normality using the Shapiro-Wilkinson normality test. None of the variables were normally distributed. Therefore, the statistical analyses were done with non-parametric tests and the data were presented with median, minimum and maximum (range). In case of paired data the Wilcoxon Rank Sum test was used. The Mann Whitney U test was used when the data were unpaired. Hypotheses were assumed when they were statistically significant (*P* ≤ 0,05).

## Results

### Study population

A total of 90 women were referred to the MMC. However, two women did not meet the criteria for menstrual (related) migraine according to ICHD-III criteria (one woman suffered from MOH, the other woman suffered from attacks of dizziness instead of headache). After performing the descriptive statistics, the sample size was further reduced to 80 women (8 women were not motivated for the multidisciplinary treatment because of pregnancy, psychological problems and other medical conditions). Of 7 women no follow up data were available. The data from 73 women were used in inferential statistics to compare the multidisciplinary approach with the previous treatment.

### Demographic and migraine characteristics

The mean age at first visit was 38.3 years (SD ± 9385) in the intervention group, and 39.59 years (SD ± 8427) in the control group. In both groups more than 75% of the women reported a family history positive for headache. The median HIT score at baseline was 65 in the intervention group, and also 65 in the control group. For more baseline characteristics, see Table 1.

**Table 1:** Baseline characteristics of patients, n (%)

	Intervention group *N* = 88	Control group *N* = 27
Years with headache at first visit		
Mean ± SD	18,66 ± 10,780	19,22 ± 9,736
Headache frequency		
1–4 per year	1 (1,1%)	-
5–11 per year	2 (2,3%)	3 (11,1%)
1 per month	31 (35,2%)	6 (22,2%)
2–4 per month	28 (31,8%)	10 (37%)
1–2 per week	18 (20,5%)	7 (25,9%)
3–5 per week	4 (4,5%)	1 (3,7%)
Daily	1 (1,1%)	-
Unknown	3 (3,4%)	-
Aggravation of headache by physical activity		
Yes	56 (63,6%)	20 (74,1%)
No	32 (36,4%)	7 (25,9%)
Aura characteristics^a^		
Yes	19 (21,6%)	6 (22,2%)
Visual symptoms	14 (15,9%)	6 (22,2%)
Speech disturbance	7 (8%)	3 (11,1%)
Sensory symptoms	9 (10,2%)	2 (7,4%)

^a^More than one answer was possible

### Comparison of outcomes between mono- and multidisciplinary treatment

During follow-up the median HIT score in the patients treated at the MMC significantly decreased from 65 at baseline to 59 points (*p* < 0,001). In the control group there was also a statistically significant decline in HIT score, from a median of 65 points at baseline to a median of 63.5 points at evaluation (*p* = 0,005).

The intervention group reported at baseline a median of 6 headache-days per month, after 9 months this was significantly reduced to a median of 3.83 days (*p* = 0,002). There was no improvement in headache-days in the control group. The median number of days with headache in the control group actually increased from 6 to 6.5 days per month.

In the intervention group the median number of days using pain medication against migraine declined significantly during follow-up. At first visit the women used pain medication at a median number of 5 days per month, and after 9 months this was declined to 3 days per month (*p* = 0,003). In the control group this variable was only included at evaluation and resulted in a median of 5 days at evaluation.

The satisfaction about treatment improved in the intervention group. At first visit the median level of satisfaction was three (sometimes satisfied with treatment), while during follow-up the satisfaction was scored with a median of four (very often satisfied). The median level of satisfaction at evaluation in the control group was also four. The multidisciplinary approach did not improve the total score of the SF12 questionnaire.

### Overview of treatments control group

In the end, 74.1% of the women with menstrual migraine who visited the headache clinic (control group) were referred to a gynaecologist. Apparently, most women in the control group needed a multidisciplinary treatment. The majority of patients in the control group received, in addition to migraine medication prescribed by a neurologist, a hormonal treatment (22 of the 27 patients) which was prescribed by a gynaecologist.

## Discussion

The treatment of patients with menstrual migraine and menstrual related migraine at a MMC gave better results compared to a mono disciplinary approach. During the multidisciplinary treatment the median HIT score improved, there was decline in headache days, and patients were using less pain medication compared to standard care. It is striking that 20 of the 27 patients from the control group were referred to a gynaecologist in course of time. Thus, 74.1% of the control group who initially received a mono-disciplinary treatment, eventually required a multidisciplinary treatment. Referring a patient with menstrual or menstrual related migraine immediately to the multidisciplinary clinic (MMC) will probably result in a more adequate and effective treatment. A multidisciplinary approach is also patient friendly as the patient does not need to visit two different clinics for the same disorder. Instead of working separately, at the menstrual migraine clinic practitioners of both disciplines can communicate, discuss and combine their knowledge resulting in a more adequate treatment.

In this study both the intervention group and the control group had a mean age of around 39 years old, varying from 15 to 55 years old. The mean and range in age is comparable with other studies [2, 12–14]. According the ICHD-III criteria menstrual migraine is usually without aura. However, a retrospective clinic-based study and another questionnaire-based study mention migraine with aura occurring menstrually, but further clinically details were lacking [3, 15]. In this study 21,6% of the intervention group and 22,2% of the control group reported aura characteristics. The explanation for the relatively high incidence of aura symptoms may lie in the fact that the questionnaires were filled in by the patient herself, and thus represents a subjective view of the migraine characteristics. There could have been confusion with symptoms which actually belong to the prodromal phase being assigned to be aura [1, 16]. Other studies also mention that women with menstrual migraine report having aura symptoms. In these studies women with menstrual related migraine experience aura symptoms. Migraine attacks around menstruation were always without aura, while the attacks at other times were associated with aura symptoms [1, 16]. In this study the patients were not asked whether their aura symptoms occurred during the menstruation or at other times of the cycle.

This study has some limitations. As it is a retrospective study we were dependent on the data which were acquired in the past. Not all questionnaires were fully completed. We tried to retrieve the data by searching in the referral letters, and in the electronic hospital record. The control group in this study included women with menstrual migraine and menstrual related migraine who were seen before 2012, during this period there was no multidisciplinary clinic for menstrual migraine. The evaluation of the mono-disciplinary approach they received before 2012, was evaluated when the menstrual migraine clinic was already started. The time interval between initiating treatment and evaluation by using the questionnaires showed up to be on average 19 months. It would have been better if the evaluation in the control group also took place after 3, 6 and 9 months. However, even with a different time interval between baseline and evaluation, there are convincing results that the multidisciplinary approach is superior to the mono-disciplinary approach. Unfortunately, a number of variables could not be compared because there was a lack of data. Finally, it should be mentioned that not all patients received the same drug therapy. For each patient was determined what was the best choice as regard to therapy. The choice of medication, both neurologically and hormonally, differed per patient. However, the aim of the study was not to treat all women with the same medication, but to compare the mono-disciplinary approach with the new multidisciplinary approach.

The comparison between our intervention group and control group is valid, because there were no significant differences in headache characteristics between both groups. Another strength of this study was the certainty of the diagnosis of menstrual migraine, because headache diaries were checked before treatment was started. However, the results of our study should be interpreted with caution as we performed a retrospective study with a relative small control group. In the future a prospective study could be performed to confirm the benefits of a multidisciplinary treatment in women with menstrual (related) migraine.

## Conclusions

It is important to treat women with menstrual and menstrual related migraine adequate, as the impact of migraine on daily life is high. Our study showed that the multidisciplinary approach is best for treating menstrual migraine. It resulted in a reduction of headache days per month, the need for pain medication and the headache impact score compared to a mono-disciplinary approach. Furthermore, it appeared that many of the patients with menstrual migraine needed a multidisciplinary treatment, as many patients were referred to a gynaecologist after visiting the neurologist. Providing a multidisciplinary approach in first instance, an adequate treatment is given directly resulting in an optimal treatment of patients with menstrual migraine.
